# New Trends in Thyroid Malignancy: Minimally Invasive Thermal Ablation Percutaneous Techniques for T1 Papillary Thyroid Carcinomas

**DOI:** 10.3390/curroncol32080442

**Published:** 2025-08-07

**Authors:** Pierre Yves Marcy

**Affiliations:** IMASUD Diagnostic and Interventional Radiology Group, PolyClinique les Fleurs, Quartier Quiez, ELSAN MEDIPOLE SUD, 83189 Ollioules, France; brozpy@gmail.com or pierre-yves.marcy@imasudradiologie.fr

During the late 1990s, thyroid nodule management strongly improved with the development of high-frequency ultrasound (HFUS) and US-guided percutaneous procedures. This allowed professionals to assess the skin surface of the neck up to five centimeters deep, including regions such as the thyroid gland, the superficial lymph node areas, and the jugular vein network. Rapidly, this led to the development of procedures to evaluate various conditions such as inflammatory infectious diseases, as well as benign and malignant conditions. Diagnosis of a benign, even giant, thyroid cyst rapidly became very easy and led to the introduction of percutaneous ethanol injection as a definitive first step in condition management, thus ruling out the need for surgical lobectomy in 99.9% of cases. In 1965, in his experimental work, McGuff reported on the potential therapeutic effect of light amplification by stimulated emission of radiation (so-called “LASER”) on thyroid carcinomas in hamsters [1]. Thirty years later, a decisive technical innovation was first reported in Italy by endocrinologist Enrico Papini and interventional radiologist CM Pacella, which was progressively built upon by R Valcalvi et al. and the South Korean team led by JH Baek, recent Italian teams led by Mauri et al as well as other smart teams who we cannot cite here: thyroid nodule thermal ablation [2,3,4,5]. The rapid development of HFUS transducers in the 1990s highlighted a novel US semiology in the field of neck pathologies and the thyroid nodule. This was necessary given the very high incidence of thyroid “incidentalomas”, which had been reported in up to 42% of healthy patients over 50 years of age in neck US daily practice [6]. In 2003, Kim et al., from Yonsei University College in Seoul, reported on the findings during that time period, namely the typical four US features (hypoechogenicity, taller than wide shape, irregular contours, and microcalcifications) of the suspicious subclinical thyroid nodule [7]. This publication still remains a key milestone of thyroid nodule assessment. This definitely paved the way for the risk stratification system assessment of thyroid nodules [8], which gave a pivotal role to US-guided fine-needle aspiration cytology (FNAC) and further molecular tests. Papillary thyroid carcinoma (PTC), the most common malignancy of the endocrine system, accounts for almost 85% of global thyroid malignancies [9]. As PTC is characterized by typical cytological features, it is most often classified as Eu-TIRADS 4 or 5 and accurately diagnosed by US-guided FNAC, being graded B5 (suspicious for malignancy) or B6 (malignant) according to the Bethesda system assessment [10]. PTC presenting a long axis < 20 mm is categorized as T1, and microcarcinoma (mPTC) is defined as a T1a nodule < 10 mm. Despite an increasing incidence, PTC still exhibits an exceptionally good prognosis, with a 99% disease-free survival rate at ten years of follow-up [9]. Concerning thyroid cancer management, American and European guidelines recommend surgical lobectomy as the appropriate therapeutic intervention [11,12]. Nevertheless, thyroid surgery carries inherent risks and drawbacks, including temporary or permanent laryngeal nerve palsy (1%), hypothyroidism (1%), temporary or permanent hypoparathyroidism, hematoma/local sepsis, lymphocele, Horner syndrome, tracheal necrosis, and some unesthetic wide scars on the neck. Therefore, alternative approaches such as active surveillance, recommended by the Japanese thyroid association, and US-guided minimally invasive ablation techniques (MITs) have been proposed for selected patients presenting with thyroid neoplasms who do not meet surgical criteria or who refuse surgery [5,13,14,15] (Table 1).

As a matter of fact, active surveillance contributes to a reduction and delay of surgical risks but also may increase, in European countries, anxiety and distress in patients concerned about potential local tumor growth, spreading of the lymph node tumor, and distant metastases. Thus, the best compromise between choosing an “aggressive surgical treatment” (with greater morbidity on an in-patient basis) or “active surveillance” (conservative treatment with the risks of delayed treatment) is indeed represented by MIT procedures (Figure 1, [5], Video S1). They now include different energy sources including laser (LA, optical fibers), radiofrequency (RFA, alternating electric current and needle), and microwave ablation (MWA, antenna needle), which combine effective tumor heating, conservative treatment of the thyroid gland, performed on an out-patient basis, protection of the patient’s peace and well-being, and significantly reduced morbidity with better cost-effectiveness and a lower complication rate [5,16,17,18] (Table 1).

Moreover, in case of the MIT’s failure, subsequent surgical management still remains available. Recently, Jeong et al. reported on the excellent long-term efficacy and safety of RFA in low-risk mPTC patients with more than ten years of follow-up [17]. However, there are currently limitations to the literature data supporting these novel techniques. At present, there is still a significant lack of multi-center phase III trials to clearly define the role of MITs in comparison to thyroid surgery and active surveillance of T1 PTC.

The encouraging results of MITs should not diminish the importance of the crucial considerations needed to obtain successful and safe results: first, selecting the right patient; second, following the guidelines provided by interventional radiologists and endocrinologists [18]; and third, finding an experienced and very skilled operator. As such, precise knowledge of the neck anatomy and a long experience of US diagnosis and percutaneous procedures in the neck are definitely needed. At least one year of graduate technical and scientific education in MIT practice and workshops should be mandatory for junior operators [19]. The current state of the art in percutaneous MITs for thyroid nodule treatment includes large local anesthesia, continuous protective cooled hydrodissection, the in-plane trans-isthmus percutaneous approach, real-time needle US monitoring, and the moving-shot technique. According to the new paradigm of patient management [20], which understands that as a patient is aging, they desire less invasiveness with more comfort, less hospitalization, and less medication, a subtle shift towards distraction-based methods has occurred. Sophrology, hypnosis, and virtual helmets provide nonpharmacological means to reduce painful experiences and anxiety [21,22]. Thus, instead of being under general anesthesia, the patient will be immersed in an “ideal world” during the procedure, acting himself with the operator for a better interactive procedure. Moreover, coughing (to prevent tracheal injury), pain manifestations (to prevent overheating or any related complications), and voice changes (which can lead to per-procedural cooled hydrodissection) are all early warning signs that an early efficient management procedure is needed.

To conclude, using MITs for mPTC is effective and safe in selected low-risk mPTC patients and integrates perfectly in a more tailored and individualized approach to thyroid cancer patient care.

A tight collaboration between endocrinologists, interventional radiologists, general practitioners, nuclear medicine physicians, and thyroid surgeons will definitely open the gate to the “Via Appia Moderna” of MIT management for T1 papillary thyroid carcinomas. This is the hope of AFTHY [23].

**Table 1:** Highlights of mPTC management: pros and cons [24,25,26].

PROCEDURE	Advantages	Drawbacks
THYROID SURGERY	Complete removal of the lobe and tumor	In-patient hospitalization (a) Risk of hypothyroidism (b) Risk of hypoparathyroidism (c) Dysphonia (d) Hematoma (e) Wound sepsis (f) Lymphocele (g) Wide neck scar (h) Horner syndrome (i) Tracheal necrosis
ACTIVE SURVEILLANCE	No intervention	Risk of active tumor progression and tumor spread
MIT LA, RFA, MWA	-Thyroid and parathyroid gland preservation -No wide neck scar -Shorter procedure -Out-patient hospitalization -LA: small localized tumors -RFA: widely used, safe and effective in PTC <10 mm -MWA: may achieve faster ablation in larger vascularized tumors	-Treated nodule remains on site -No lymph node control -Nodular rupture (2%) -Local (skin, trachea) burns -(c,d,e,h,i) MIT < surgery

Complications (a), (b), and (f) are not encountered in MIT procedures; nodule rupture [27] and local burns [28] are specific complications related to MIT; and complications (h) and (i) are reported as exceptional in both surgery and MIT.

## Figures and Tables

**Figure 1:**
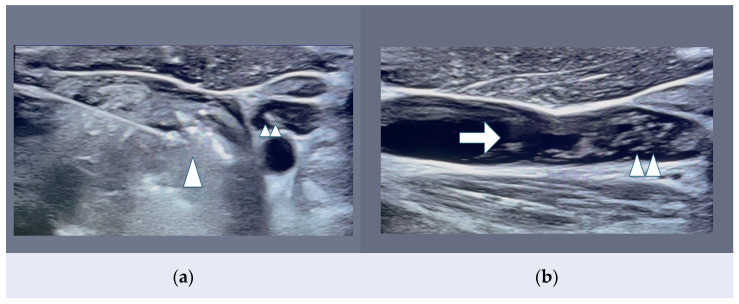
(**a**,**b**) MWA visual thermal effect of left thyroid nodule. Echogenic (bright) effect represents thyroid nodule tissue heating during the moving-shot technique; the treating antenna needle is gradually retrieved as echogenic therapeutic effects appear on the US in-plane scan. Figure (**b**) displays gas bubbles draining into the ipsilateral internal jugular vein (IJV). (**a**) Treatment with an antenna needle during MWA procedure. Axial horizontal US scanning of left thyroid nodule showing cloudy effect (arrowhead) appearing at the needle tip, and gas bubbles (arrowheads) draining into the left IJV. (**b**) Vertical US scanning along left internal jugular vein (IJV). MWA-related gas bubbles (arrowheads) are shown moving with the internal jugular vein (IJV) flow (arrow) into the right heart cavities.

## Supplementary Materials

The following supporting information can be downloaded at https://www.mdpi.com/article/10.3390/curroncol32080442/s1, Video S1: MWA thermal procedure under real-time ultrasound (US) guidance. Horizontal US scanning of left thyroid nodule being treated by using a trans-isthmic MWA needle. Echogenic foci related to thermal “cloudy” effect are produced at the needle tip. US probe is then rotated 90° along the long axis of the IJV, as gas bubbles are detected flowing down to the right heart cavities.

## Abbreviations

MIT: minimally invasive therapy; LA: laser ablation; RFA: radiofrequency ablation; MWA: microwave ablation; PTC: papillary thyroid carcinoma; US: ultrasound; FNAC: fine-needle aspiration cytology; IJV: internal jugular vein.

## References

[1] Pacella C.M., Bizzarri G., Guglielmi R., Anelli V., Bianchini A., Crescenzi A., Pacella S., Papini E. (2000). Thyroid tissue: US-guided percutaneous interstitial laser ablation-a feasibility study. Radiology.

[2] Valcavi R., Piana S., Bortolan G.S., Lai R., Barbieri V., Negro R. (2013). Ultrasound-guided percutaneous laser ablation of papillary thyroid microcarcinoma: A feasibility study on three cases with pathological and immunohistochemical evaluation. Thyroid.

[3] Baek J.H., Lee J.H., Valcavi R., Pacella C.M., Rhim H., Na D.G. (2011). Thermal ablation for benign thyroid nodules: Radiofrequency and laser. Korean J. Radiol..

[4] Mc Guff P.E., Deterling R.A., Gottlieb L.S., Fahimi H.D., Bushnell D., Robert F. (1964). The Laser treatment of experimental malignant. tumors. Can. Med. Assoc. J..

[5] Mauri G., Orsi F., Carriero S., Della Vigna P., De Fiori E., Monzani D., Pravettoni G., Grosso E., Manzoni M.F., Ansarin M. (2021). Image-Guided Thermal Ablation as an Alternative to Surgery for Papillary Thyroid Microcarcinoma: Preliminary Results of an Italian Experience. Front. Endocrinol..

[6] Bruneton J.N., Balu-Maestro C., Marcy P.Y., Melia P., Mourou M.Y. (1994). Very high frequency (13 MHz) ultrasonographic examination of the normal neck: Detection of normal lymph nodes and thyroid nodules. J. Ultrasound Med..

[7] Kim E.K., Park C.S., Chung W.Y., Oh K.K., Kim D.I., Lee J.T., Yoo H.S. (2002). New sonographic criteria for recommeding fine-needle aspiration biopsy of nonpalpable solid nodules of the thyroid. Am. J. Roentgenol..

[8] Kim D.H., Kim S.W., Basurrah M.A., Lee J., Hwang S.H. (2023). Diagnostic Performance of Six Ultrasound Risk Stratification Systems for Thyroid Nodules: A Systematic Review and Network Meta-Analysis. Am. J. Roentgenol..

[9] Baloch Z.W., Asa S.L., Barletta J.A., Ghossein R.A., Juhlin C.C., Jung C.K., LiVolsi V.A., Papotti M.G., Sobrinho-Simões M., Tallini G. (2022). Overview of the 2022 WHO Classification of Thyroid Neoplasms. Endocr. Pathol..

[10] Cochand-Priollet B., Maleki Z. (2023). Cytology and Histology of Thyroid Nodules: Exploring Novel Insights in the Molecular Era for Enhanced Patient Management. Curr. Oncol..

[11] Sawka A.M., Carty S.E., Haugen B.R., Hennessey J.V., Kopp P.A., Pearce E.N., Sosa J.A., Tufano R.P., Jonklaas J. (2018). American Thyroid Association Guidelines and Statements: Past, Present, and Future. Thyroid.

[12] Kanona H., Virk J.S., Offiah C., Stimpson P. (2017). Ultrasound-guided assessment of thyroid nodules based on the 2014 British Thyroid Association guidelines for the management of thyroid cancer-how we do it. Clin. Otolaryngol..

[13] Lee J.Y., Na D.G. (2025). Ultrasound Imaging Criteria and Protocols for Active Surveillance of Low-Risk Thyroid Cancer: A Review of International Consensus Guidelines. Endocrinol. Metab..

[14] Ghai S., O’Brien C., Goldstein D.P., Sawka A.M., Canadian Thyroid Cancer Active Surveillance Study Group (2021). Ultrasound in active surveillance for low-risk papillary thyroid cancer: Imaging considerations in case selection and disease surveillance. Insights Imaging.

[15] Marcy P.Y., Russ G., Saba L., Sanglier J., Ghanassia E., Sharara H., Thariat J., Morvan J.B., Bizeau A. (2022). Opinion: Leading position of ultrasound in decision algorithm for small papillary thyroid carcinoma. Insights Imaging.

[16] Zhou W., Jiang S., Zhan W., Zhou J., Xu S., Zhang L. (2017). Ultrasound-guided percutaneous laser ablation of unifocal T1N0M0 papillary thyroid microcarcinoma: Preliminary results. Eur. Radiol..

[17] Jeong S.Y., Baek S.M., Shin S., Son J.M., Kim H., Baek J.H. (2025). Radiofrequency Ablation of Low-Risk Papillary Thyroid Microcarcinoma: A Retrospective Cohort Study Including Patients with More than 10 Years of Follow-up. Thyroid.

[18] Mauri G., Hegedüs L., Bandula S., Cazzato R.L., Czarniecka A., Dudeck O., Fugazzola L., Netea-Maier R., Russ G., Wallin G. (2021). European Thyroid Association and Cardiovascular and Interventional Radiological Society of Europe 2021 Clinical Practice Guideline for the Use of Minimally Invasive Treatments in Malignant Thyroid Lesions. Eur. Thyroid J..

[19] EuroMITT International Congress on Minimally Invasive Thyroid Treatments. https://eumitt.com/mitt-2025/.

[20] Monpeyssen H. (2023). Transitioning from Traditional Academic Decision Making to Patient-Centric Healthcare Choices: The Example of Thyroid Thermal Ablation Techniques for Papillary Thyroid Microcarcinomas. Curr. Oncol..

[21] Grange L., Grange R., Bertholon S., Morisson S., Martin I., Boutet C., Grange S. (2024). Virtual reality for interventional radiology patients: A preliminary study. Support. Care Cancer.

[22] Bertrand A.S., Iannessi A., Buteau S., Jiang X.Y., Beaumont H., Grondin B., Baudin G. (2018). Effects of relaxing therapies on patient’s pain during percutaneous interventional radiology procedures. Ann. Palliat. Med..

[23] AFTHY: Association Francophone de Thyroïdologie. https://www.afthy.fr.

[24] Gao X., Yang Y., Wang Y., Huang Y. (2023). Efficacy and safety of ultrasound-guided radiofrequency, microwave and laser ablation for the treatment of T1N0M0 papillary thyroid carcinoma on a large scale: A systematic review and meta-analysis. Int. J. Hyperth..

[25] Ren Y., Li Y., Chu X., Chen G., Han X., Zhao Y., Liu C., Wang J., Xu S. (2025). Ultrasound-guided microwave ablation versus surgery for low-risk solitary papillary thyroid microcarcinoma: A propensity-matched cohort study. Endocr. Connect..

[26] Xu X., Peng Y., Han G. (2024). Comparative efficacy of different thermal ablation and conventional surgery for the treatment of Papillary Thyroid Microcarcinoma: Systematic review including traditional pooling and Bayesian network meta-analysis. Am. J. Otolaryngol..

[27] Austerlitz J., Mann D.S., Noel J.E., Orloff L.A. (2024). Thyroid Nodule Rupture Following Radiofrequency Ablation for Benign Thyroid Nodules. JAMA Otolaryngol. Head Neck Surg..

[28] Yang X., Wang J., Tang Q., Gong R., Wei T., Li Z. (2025). Delayed Tracheal Perforation After Microwave Ablation of Papillary Thyroid Microcarcinoma: A Case Report and Literature Review. Ear Nose Throat J..

